# Machine Learning Model with Fourier-Transform Infrared Spectroscopy (FTIR) as a Proof-of-Concept Tool for Predicting Group A *Streptococcus* (GAS) *emm*-Type in the Pediatric Population

**DOI:** 10.3390/diagnostics15233041

**Published:** 2025-11-28

**Authors:** Valeria Fox, Gianluca Vrenna, Martina Rossitto, Serena Raimondi, Marco Cristiano, Venere Cortazzo, Marilena Agosta, Barbara Lucignano, Manuela Onori, Vanessa Tuccio Guarna Assanti, Maria Stefania Lepanto, Nour Essa, Isabella Tarissi De Jacobis, Andrea Campana, Massimiliano Raponi, Alberto Villani, Carlo Federico Perno, Paola Bernaschi

**Affiliations:** 1 Multimodal Laboratory Medicine, Bambino Gesù Children’s Hospital, IRCCS, 00165 Rome, Italy; 2 Microbiology and Diagnostic Immunology Unit, Bambino Gesù Children’s Hospital, IRCCS, 00165 Rome, Italy; 3 General Pediatric and Infectious Disease Unit, Pediatric Emergency Medicine, Bambino Gesù Children’s Hospital, IRCCS, 00165 Rome, Italy; 4 Pediatrics Unit, Bambino Gesù Children’s Hospital, IRCCS, 00165 Rome, Italy; 5 Medical Direction, Bambino Gesù Children’s Hospital, IRCCS, 00165 Rome, Italy

**Keywords:** FTIR, machine learning models, GAS, *emm*-type prediction

## Abstract

**Background**: Since 2022, invasive Group A *Streptococcus* (GAS) infections have increased, mainly due to the spread of specific *emm*-types, such as *emm1*. As therapy may depend on the *emm*-type, rapid and cost-effective identification is crucial. Fourier-transform infrared spectroscopy (FTIR) has emerged as a promising alternative to sequencing for GAS typing. We applied machine learning (ML) to FTIR spectra to build a predictive model for *emm*-type identification. **Methods**: Twenty-four GAS strains were analyzed by whole-genome sequencing and FTIR. The model was trained on twenty-one strains (*emm*-types: 1, 3, 4, and 6), using leave-one-out cross validation (LOOCV). To test the model’s ability to avoid false positive results, the model was also tested with three strains belonging to *emm-*types not included in the training of the model (*emm*-types: 12, 89, and 75). **Results**: An artificial neural network trained for 400 cycles achieved the highest accuracy (90.7%) out of the thirteen different models tested. When the three strains belonging to *emm-*types not included in the model were predicted with this model, it produced low score values, confirming its ability to avoid false positive results. **Conclusions**: We developed a preliminary and proof-of-concept model capable of accurately predicting the four most-prevalent *emm*-types in our setting, including the highly virulent *emm*1. These findings support FTIR combined with ML as a rapid, low-cost tool for GAS typing, with potential for real-time clinical applications to guide timely treatment decisions. However, as a proof-of-concept study, the relatively small sample size and limited *emm*-type diversity underline the need for further validation with larger and more diverse datasets.

## 1. Introduction

Group A *Streptococcus* (GAS) is an important human pathogen. It is commonly recognized as the etiological agent of scarlet fever, primarily, but not exclusively, in children. Moreover, it is responsible for a wide spectrum of disease manifestations, ranging from asymptomatic colonization and pharyngitis to high-mortality and invasive diseases [1,2,3]. After the COVID-19 pandemic, restrictive measures had resulted in a global reduction in invasive GAS (iGAS) infections, but a rapid upsurge has been observed in several countries since the end of 2022, both in pediatric and adult populations [4,5,6,7,8]. This rise has mainly been attributed to the spread of specific *emm*-types, like *emm*-type 1. In particular, an increase in *emm*-type 1, and in the M1_UK_ sub-lineage, characterized by a heightened expression of the streptococcal pyrogenic exotoxin A (SpeA), has been observed, resulting in an increase in invasive infections [9,10,11]. Given that *emm*-type and toxin expression can influence the choice of antibiotic and anti-inflammatory treatments, the rapid identification of the *emm*-type has gained importance in clinical settings. In fact, timely identification is essential not only to guide the appropriate antimicrobial therapy and implement correct infection-control measures, but the early detection of particularly virulent *emm*-types can also help in reducing morbidity and mortality rates. However, the determination of the *emm*-type is usually conducted by traditional methods, such as phenotypic characterization, or by sequencing the *emm* gene, encoding for the M protein, a technique that remains too costly and time-consuming to be routinely introduced in diagnostics [12]. Recently, Fourier-transform infrared spectroscopy (FTIR) has emerged as a fast and cost-effective alternative for microbial typing, showing great potential for being implemented in clinical practice to obtain reliable results in a short time [13,14]. In fact, IR spectroscopy is able to provide a sort of molecular fingerprint by looking in the whole mid-IR wavenumber region (4000–500 cm^−1^, with the possibility of investigating and focusing on narrower spectral regions, corresponding to the infrared radiation absorption of the different biomolecules) [15,16]. Moreover, more recently, artificial intelligence and machine learning (ML) algorithms have been combined with FTIR to identify specific spectra patterns which might go unnoticed through conventional analyses, and thus automatically and rapidly predict microbial characteristics. However, to date, the application of this combined technology (FTIR-ML) has been implemented in the typing of other microorganisms (such as *Streptococcus pneumoniae* and *Salmonella enterica*), but not in GAS typing [17,18,19]. Our study represents a first proof-of-concept strategy that explores the feasibility of this approach, which could help bridge the gap between research and routine diagnostics. In contrast to conventional methods like whole-genome sequencing (WGS) which, albeit highly informative and the gold standard, remains costly, time-consuming, and requires specialized expertise, the use of FTIR has the potential to deliver rapid and low-cost results which can be obtained even by non-expert personnel, allowing for its direct application in clinical microbiology laboratories.

Thus, the three main contributions of this study to the current literature are (i) to provide a proof-of-concept application of FTIR combined with ML for GAS *emm*-type prediction; (ii) to explore its feasibility as a rapid, cost-effective, and easy alternative to WGS in clinical settings; and (iii) to lay the groundwork for future broader and multicenter studies, including a bigger and more diverse strain collection, both in terms of *emm-*type and patient population (children and adults).

The aim of this study was to construct an internal database of *emm*-types circulating in our center and to build and validate a proof-of-concept machine learning classifier able to rapidly and reliably predict the *emm*-type of GAS strains based on their FTIR spectra.

## 2. Materials and Methods

### 2.1. Bacterial Collection and Microbial Identification

To establish the dataset for model development, Group A *Streptococcus* (GAS) clinical isolates from invasive (iGAS) and non-invasive infections in pediatric patients admitted at the Bambino Gesù Children’s Hospital in Rome between February 2023 and October 2024 were included in the study. The strategy of including both invasive and non-invasive isolates aimed to capture the diversity of clinical presentations and was chosen to develop a classifier with broad applicability in pediatric clinical microbiology settings. The isolates reflect the availability during the study period and were not selected through randomization or strategic sampling. Some of the strains (8/24, 33.3%) were already characterized by whole-genome sequencing in a previous study [20].

The culturing of GAS isolates was performed on Columbia agar plates supplemented with 5% sheep blood (bioMérieux, Marcy-l’Étoile, France) incubated at 35–37 °C overnight in a 5% CO_2_ atmosphere. Colonies grown were then identified by matrix-assisted laser desorption ionization–time-of-flight mass spectrometry (MALDI-TOF MS; Bruker Daltonics, Bremen, Germany).

### 2.2. DNA Extraction and Whole-Genome Sequencing (WGS)

As a reference method for strain characterization, we performed WGS to determine the *emm*-type and to discriminate M1 sub-lineages, providing the genetic background for evaluating the FTIR-based approach. Bacterial DNA extraction was performed using the extraction kit (EZ1&2 DNA tissue kit, Qiagen, Hilden, Germany) on the automatic extractor EZ1 (BioRobot EZ1, Qiagen, Hilden, Germany), following the manufacturer’s instructions, with the elution volume set at 50 µL. The obtained DNA was then quantified with a Qubit fluorometer (Qubit^®^ dsDNA HS Assay Kits, Thermo Fisher Scientific, Waltham, MA, USA), and sequencing library preparation was performed according to the manufacturer’s protocol with a DNAprep kit (Illumina, San Diego, CA, USA). Prepared libraries were sequenced with an Illumina NextSeq 550 sequencing platform using a NextSeq 500/550 v2.5 Kit in paired-end mode (150 × 2). 

Raw reads were filtered for quality (Phred score > 28) and the presence of adapters by Fastp (v.0.23.4) [21] and then checked with FastQC (v0.11.9) [22] and MultiQC (v.1.18) [23]. Kraken2 (v1.1.3) [24] with the standard database was used to taxonomically classify reads and screen for the presence of potential contamination. A *de novo* assembly was then performed using Shovill (v1.1.0) [25] and the quality of assembled contigs was checked through Quast (v5.1) [26]. Assembled contigs were used for *emm*-typing and multilocus sequence typing (MLST) prediction, performed with the *emm*-typer (v.0.2.0) [27] and mlst (v2.23.0) [28], respectively. *Emm*1 sub-lineages were investigated by looking at the single-nucleotide polymorphisms (SNPs) obtained with Snippy (v4.6.0) [29], using the *S. pyogenes* MGAS5005 genome (GenBank accession number NC_007297) as reference, as previously described, to distinguish the M1_UK_ to the M1_global_ sub-lineage by looking at the presence of the 27 SNPs characteristic of the M1_UK_ lineage [30].

### 2.3. Fourier-Transform Infrared Spectroscopy (FTIR) Sample Preparation and Spectra Acquisition

In parallel to WGS, isolates were analyzed by FTIR spectroscopy to obtain reproducible spectral fingerprints that could be used for exploratory clustering and subsequent machine learning model creation. FTIR analysis was performed by the IR Biotyper system (IRBT—Bruker Daltonics GmbH & Co. KG, Bremen, Germany). Isolates were refreshed and incubated at 35–37 °C on a Columbia agar plate supplemented with 5% sheep blood (bioMérieux, Marcy-l’Étoile, France) in a 2.5 L CO_2_ gen pack (ThermoFisher Scientific, Waltham, MA, USA). GAS strains were prepared for FT-IR spectroscopy using the direct smear method, with some modifications [31]. Briefly, a 1 μL plastic loop was used to carefully collect the biomass from the plate and was evenly applied to the IRBT silicon plate containing 96 spots (Bruker, Bremen, Germany) using a single drop of distilled water. The same plastic loop was then used to carefully spread the drop of water with the biomass to obtain a homogeneous suspension. In addition to the GAS strains, two *E. coli* reference strains (IRTS 1 and IRTS 2; Bruker) were also included on the plate as quality control, to validate the run. Once the samples were completely dried under the laminar flow cabinet, the silicon plate was exposed to a 25 W UV lamp, located in the same laminar flow cabinet, to inactivate the bacterial cells. To minimize batch effects and other technical variations that might impact the FTIR spectral data, all isolates were cultured and prepared under strictly standardized conditions regarding medium composition, incubation temperature, CO_2_ atmosphere, and incubation times. Sample preparation steps for FTIR, including biomass collection, suspension homogenization, drying, and UV inactivation, were consistently applied. Furthermore, internal instrument controls and routine calibrations inherent to the IR Biotyper system helped ensure reproducibility across runs.

### 2.4. Exploratory Analysis and Machine Learning Classifier Creation

Upon FTIR spectra acquisition, a first exploratory analysis was performed to investigate the clustering ability of the different *emm*-types and thus the possibility of creating classifiers to accurately discriminate between them. The spectral window between 1300 and 800 cm^−1^ was chosen as it corresponds to the fingerprint region in FTIR spectroscopy specifically related to carbohydrate absorption. This region is biologically relevant and has been shown to provide the best discrimination between *emm*-types compared to other spectral ranges tested. A dendrogram was constructed using the default splicing method (1300–800 cm^−1^) and the Ward method based on Euclidean distance, grouping the average spectra for each isolate. Dimension reduction was performed by first applying principal component analysis (PCA) to reduce the original data to 30 principal components (PCs), capturing most of the variance. Then, linear discriminant analysis (LDA) was applied, resulting in 20 linear discriminants (LDs), which explained 98.3% of the total variance. The spatial distribution of the different *emm*-types was also observed at the scatter plots in 2D and 3D, using PCA and LDA with the default splicing method. The deviation plot was also used to assess the differences between samples and thus determine the number of components to be used in the algorithms for model creation. A first analysis was performed dividing the two *emm*1 sub-lineages detected (M1_UK_ and M1_global_), which were then merged into a single *emm1* group, since no specific clustering pattern able to discriminate between the two lineages could be observed.

The IR Biotyper software (v4.0) was used to create a ML classifier able to identify the *emm*-types most represented in our setting (*emm*1, *emm*3, *emm*4, and *emm*6). The IR Biotyper software (Bruker) allows the user to select among different algorithms (ANN, SVM, and RBF) and to adjust a defined set of parameters (e.g., number of training cycles, C value, and gamma).

Given the low number of isolates available, we decided to apply a leave-one-out cross validation (LOOCV) approach to evaluate the performance of the different classifiers and to minimize the risk of overfitting. More specifically, each isolate was iteratively used as a test sample while all remaining isolates were used to train the model. This process was then repeated until all isolates served once as the validation set. Thus, a total of 21 strains, belonging to the 4 *emm*-types 1, 3, 4, and 6, were used to construct the model (Table 1). The 3 strains belonging to *emm*-types not included in the model (*emm*12, *emm*75, and *emm*89) were retained in the test set in order to evaluate the classifier’s ability to avoid false positive assignments.

Several classifiers were built using different algorithms (i.e., artificial neural network, ANN, linear support vector machine, SVM, and radial basis functions, RBFs) with different parameters (i.e., number of training cycles, C value, and gamma) using the same training set. Model performance was evaluated by averaging the classification results across all iterations, and accuracy was calculated as the ratio of correctly classified spectra to the total number of spectra. To assess the reliability of the model’s predictions, we further calculated classification metrics including sensitivity (recall), specificity, precision, and the F1-score. These metrics provide a comprehensive evaluation of the model’s ability to correctly identify true positives and true negatives, as well as its robustness in minimizing false positive and false negative assignments. The reliability scores reflect these combined performance aspects, indicating confidence in the classifier’s predictions at both the spectrum and isolate levels.

The overall workflow described above is summarized in Supplementary Figure S1.

## 3. Results

### 3.1. GAS Strains Description 

A total of 24 clinical strains causing both invasive and non-invasive infections were isolated from different materials, based on strain availability during the study period. The characteristics of the clinical strains and their use in the ML model are detailed in Table 1.

*Emm*-typing revealed the presence of a total of seven *emm*-types (Table 1). Among these, three *emm-*types (*emm*12, *emm*75, and *emm*89) consisted of only one strain each, but were still retained in the test set in order to evaluate the classifier’s ability to avoid false positive assignments. Moreover, among the nine *emm*1 strains, five (55.6%) belonged to theM1_UK_ and four (44.4%) to the M1_global_ sub-lineages.

### 3.2. Exploratory Analysis

**Figure 1:**
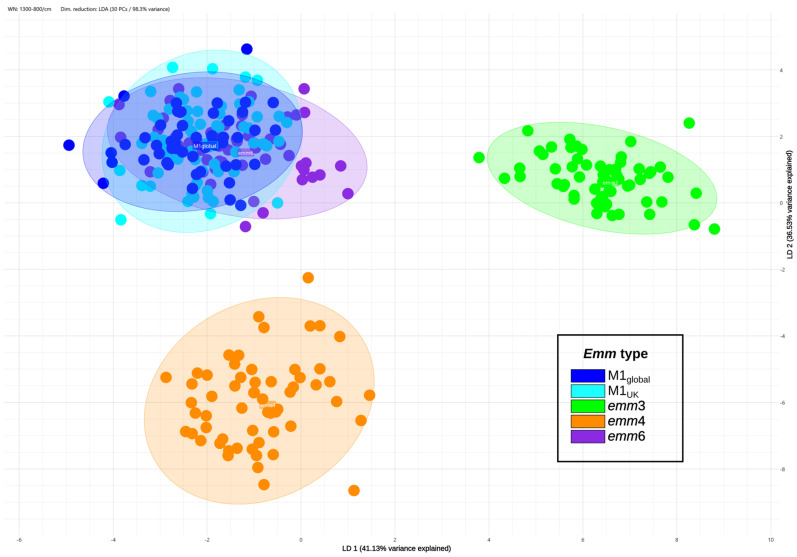
2D scatter plot based on linear discriminant analysis (LDA) of the spectra. Spectra are colored based on *emm*-type, with a distinction between the *emm*1 sub-lineages M1_UK_ and M1_global_, which was not taken into consideration in the training of the model, where they were all treated as *emm*1. The percentage of variance explained by each LDA axis is indicated on the corresponding axis.

The spatial distribution of the spectra in the 2D scatter plot obtained with LDA showed clustering of spectra based on the *emm*-type, although no specific clustering could be observed for the M1_UK_ and M1_global_ lineages (Figure 1). For this reason, it was decided to consider only the *emm*1 lineage together for the model construction, without dividing the M1_UK_ and M1_global_ lineages.

**Figure 2:**
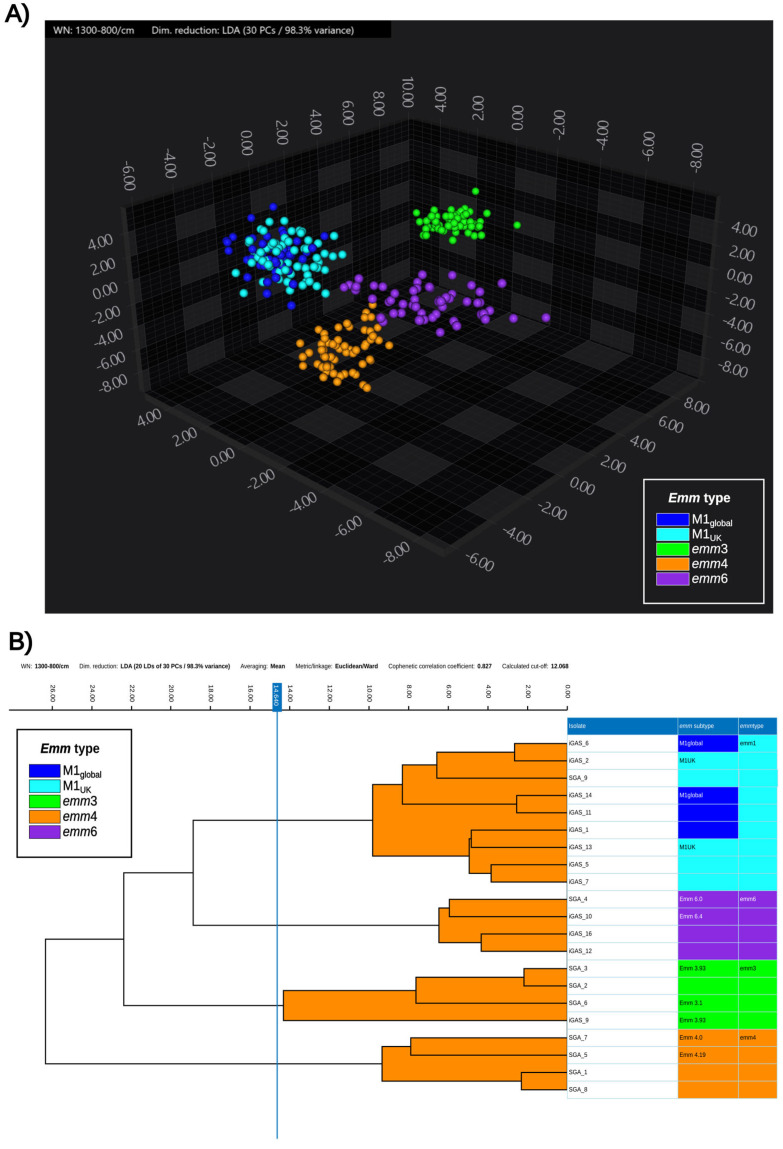
3D scatter plot (**A**) and hierarchical clustering dendrogram (**B**) of the spectra from the 21 isolates included in the training and validation dataset. (**A**) Spectra are colored according to the *emm*-type. The first three principal components out of the eleven accounting for 98.6% of the variance are shown. (**B**) Hierarchical cluster analysis performed with Ward’s method based on Euclidean distance, grouping the average spectra for each isolate. Both the *emm*-subtype and the *emm-*type are reported on the right side of the figure, next to the isolate’s name, and in the legend. The blue line represents the automatic cut-off calculated by the software to discriminate clonal strains.

While *emm*3 and *emm*4 were clearly separated, only a marginal overlap could be seen between the spectra of *emm*1 and *emm*6, which was not considered to hinder efficient discrimination among the different *emm*-types. The same distribution could be observed at the 3D scatter plot (Figure 2A) and the dendrogram (Figure 2B).

### 3.3. Classifiers Evaluation

Among all the models tested, the most accurate was an artificial neural network (ANN) model, trained for 400 cycles, which resulted in an overall accuracy of 90.7%, a sensitivity of 89.9%, specificity of 98.2%, precision of 94.4, and an F1-score of 0.92 (Supplementary Table S1).

A list of all the algorithms and parameters tested, with the relative accuracy and error rate values, divided by *emm-*type, are listed in Supplementary Table S1.

The model was then tested with the test dataset, which included three strains from *emm*-types not used in model training to evaluate false positive prediction rates. Supplementary Figure S2A shows the Bruker report excerpts indicating that although the model generated tentative classifications for these isolates, the low reliability scores signaled poor confidence in these assignments, effectively reducing false positive calls. Supplementary Figure S2B presents the confusion matrix of the test dataset, illustrating the misclassification patterns among these foreign *emm*-types. In particular, most *emm*12 spectra were misclassified mainly as *emm*1, followed by *emm*6, while *emm*75 and *emm*89 spectra also showed misclassification trends toward *emm*4, *emm*6, and *emm*1. These results confirm that while the model occasionally assigns known *emm*-types to unknown isolates, the low scores provide a safeguard to minimize incorrect confident predictions, supporting the model’s robustness in distinguishing unknown *emm*-types.

## 4. Discussion

Obtaining a rapid and accurate diagnosis is one of the most critical aspects of clinical management in infectious diseases, especially in vulnerable populations such as infants and neonates. In these patients, infections can progress extremely rapidly, and any delays in diagnosis may lead to severe complications or even death. A timely and precise identification of the causative agent is therefore essential to initiate targeted therapy as early as possible, ultimately improving clinical outcomes and reducing mortality rates [32,33]. This is particularly crucial in the case of the highly virulent Group A *Streptococcus* (GAS), which can cause severe and rapidly progressing infections [1,3]. Thus, the fast identification of these strains is essential to initiate the appropriate antibiotic and anti-inflammatory treatments in a timely manner. In recent years, it has been observed that specific *emm*-types hold higher pathogenic potential compared to other *emm*-types [3,34]. For this reason, being able to rapidly distinguish between *emm*-types is important for guiding targeted treatment and infection-control strategies.

Nonetheless, this precise and rapid diagnostic process often requires significant costs, time, and the need for specialized laboratory infrastructures. Whole-genome sequencing (WGS), for instance, although highly informative and the gold standard for most applications, is time-consuming and requires specific equipment and skilled personnel for both wet-lab processing and bioinformatic analysis. In addition, the time to achieve the result with WGS is relatively long and therefore not always compatible with the natural clinical evolution of the disease; thus, it has more of a scientific (post hoc) rather than clinical relevance. These limitations make its implementation in routine clinical settings challenging, especially in low-resource environments.

In contrast, both Fourier-transform infrared spectroscopy (FTIR) and matrix-assisted laser desorption ionization–time-of-flight (MALDI-TOF) mass spectrometry offer faster and more cost-effective alternatives for bacterial typing. In fact, in recent years, FTIR technology has emerged as a promising alternative to WGS for microbial typing, due to its rapid, cost-effective, and easy-to-use approach [35]. Apart from microbial typing, this technology has also been used for other applications, including the use of machine learning (ML) for the creation of models able to predict specific microbial characteristics [36,37]. Even if MALDI-TOF is widely established in clinical laboratories and typically provides quicker species-level identification, its capacity to develop and deploy machine learning models for automated strain typing accessible to routine clinical technicians is often challenging. In the IR Biotyper software, instead, the possibility for creating machine learning models is directly integrated into the user-friendly software platform, facilitating rapid and accurate strain-level discrimination by frontline laboratory staff. Thus, FTIR strikes a practical balance between accuracy, speed, cost, and ease of use, presenting a valuable tool for rapid bacterial typing in clinical microbiology alongside MALDI-TOF and WGS.

While there is a study applying FTIR to Group A *Streptococcus*, it primarily addresses outbreak identification and cluster discrimination rather than molecular *emm*-typing [38]. In this study, instead, we explored the potential of FTIR combined with ML to rapidly and accurately predict the *emm*-type of GAS isolates, in order to rapidly identify strains more associated with invasive infections and guide clinicians in the choice of the appropriate therapy. This FTIR-ML approach also holds significant potential to positively impact antibiotic stewardship efforts. Rapid and precise identification of GAS *emm*-types associated with invasive disease allows clinicians to tailor antibiotic therapy more effectively, potentially reducing unnecessary broad-spectrum antibiotic use. Early detection of high-risk strains supports timely initiation of appropriate targeted therapy and limits overtreatment, which is crucial to combating antibiotic resistance. By enabling fast turnaround times compatible with clinical decision-making, our method can contribute to improved antimicrobial stewardship and better patient outcomes, especially in vulnerable populations such as infants and neonates.

This approach holds clinical relevance, supported by the rapid turnaround time of FTIR spectroscopy. Indeed, spectral acquisition requires only a few hours, and once the model is established, the classification of isolates is performed in seconds. This timeframe is fully compatible with the clinical evolution of invasive infections, making FTIR a promising tool not only for research purposes but also for real-time diagnostic use.

In fact, once the classifier is developed and stored in the software, it can be integrated into routine clinical practice. When a new spectrum is acquired, following standard culture and identification steps, the technician can directly apply the pre-established model to the acquired spectrum using the IR Biotyper software, receiving the predicted *emm*-type and corresponding reliability score within seconds. This rapid turnaround allows for the timely identification of high-risk *emm*-types directly in the laboratory, enabling early treatment decisions and targeted infection-control measures.

Although the isolates included in this study reflected the availability during the study period and were not selected through randomization or strategic sampling, the *emm*-types observed (*emm*1, *emm*3, *emm*4, and *emm*6) are consistent with those prevalently circulating in pediatric populations both in our clinical center and in other Italian and European settings [39,40,41], providing some indirect support for the representativeness of the dataset.

The best-performing model in our study was the artificial neural network (ANN) trained for 400 cycles, which consistently outperformed the other models tested, i.e., the support vector machine (SVM) and radial basis function (RBF) classifiers, as shown by its superior accuracy, sensitivity, specificity, precision, and F1-score metrics. ANN algorithms proved to be well-suited for handling complex, high-dimensional data such as FTIR spectra due to their ability to model non-linear relationships and perform hierarchical feature extraction. This likely enables them to capture subtle spectral variations and interactions among biochemical components that other models, like SVM and RBF, may not fully exploit. In fact, the model that we developed showed high potential, since it achieved high accuracy levels, also retaining the ability to avoid false positives when tested with strains belonging to *emm*12, *emm*75, and *emm*89, which were not included in the model. In fact, these strains were not misclassified but rather unassigned, since the classification scores were low, implying that the model is able to limit false positive predictions when used in the presence of unknown data. However, since the model output space only includes four *emm*-types, every input is assigned to one of these classes. Thus, traditional false positive control is limited as the model cannot classify isolates as “unknown.” In this context, false positives are defined as isolates from unknown *emm*-types incorrectly assigned to known classes. While the test dataset included strains from foreign *emm*-types that were correctly left unassigned due to low confidence scores, the evaluation does not quantitatively reflect false positive control in a strict sense. Future work could improve this aspect by incorporating a rejection option or an explicit unknown class in the model to better handle unseen data and rigorously assess misclassification rates.

These findings underscore once again the utility of FTIR not only for microbial typing, but also for predicting microbial features with the aid of ML. This could represent an important tool in clinical microbiology laboratories, also those in low-resource settings, especially in contexts where a fast diagnosis is essential for correct therapeutic decisions. At present, in our laboratory, FTIR is already part of the routine workflow, mainly for the investigation of potential outbreaks, when speed is crucial. The use of a model for predicting *emm-*type in GAS strains could be implemented in a similar way, directly acquiring the FTIR spectra after standard culture and MALDI-TOF strain identification. This will allow spectra to be obtained in a matter of hours and to predict the *emm-*type in just seconds, allowing the microbiologist to notify the clinicians about the possible presence of high-risk *emm-*types in actionable timeframes. This shows how the proof-of-concept described here could be translated into a rapid and effective operational step in the diagnostic routine.

Despite these promising results, this work contains some limitations. Since it represents a proof-of-concept study, the small sample size and limited variety of *emm*-types included in the model do not allow for generalization. These limitations are primarily due to the fact that we only used microbial strains isolated in our center, which inevitably restricts both the genetic diversity and the epidemiological representativeness of the dataset. To avoid overfitting in the validation of the model, we resorted to the LOOCV strategy. Nonetheless, given the limited number of samples, the point estimates of model accuracy and related metrics may not fully capture the true variability and uncertainty; thus, the use of confidence intervals would be valuable in future studies for a more rigorous uncertainty quantification. Additionally, the test set consisted of three strains that belonged to *emm*-types not included in the training of the model. Although this provided some useful insight into the classifier’s ability to minimize false positive predictions, it does not constitute rigorous external validation. To thoroughly assess the model’s robustness and its applicability to real-world scenarios, an independent dataset, ideally collected from various centers, geographical locations, and age groups, is essential. To address this limitation, a multicenter study involving multiple clinical microbiology laboratories across Italy, and potentially across different countries and age groups, could be conducted. This approach would enable the inclusion of a larger number and wider variety of *emm*-types, thereby improving the robustness and generalizability of the model. Furthermore, future research should also consider exploring advanced machine learning techniques such as transfer learning (i.e., leveraging models trained on larger datasets) and incremental learning (i.e., progressively updating the model as new isolates become available) to overcome the challenges posed by small datasets.

While we have addressed the main limitations regarding sample size and genetic diversity, it is important to emphasize the critical impact of dataset quality, representativeness, and geographic heterogeneity on machine learning model performance in clinical microbiology contexts. Larger, multi-institutional datasets incorporating broader clinical, demographic, and geographic diversity are essential to enhance model robustness and external validity. Furthermore, future work should prioritize the continuous adaptation and re-validation of models using incremental learning approaches to include evolving pathogen populations and emerging *emm*-types. Future perspectives will thus focus on expanding the strain collection and performing multicenter validation to comprehensively assess the model’s performance in different settings. Nevertheless, the main aim of this work was to provide a proof-of-concept study showing that FTIR combined with ML can be applied to GAS typing and that this approach is easily implementable in routine clinical microbiology laboratories.

## 5. Conclusions

In conclusion, in this proof-of-concept study, we analyzed 24 GAS isolates by WGS and FTIR, building an ML classifier trained on 21 strains with the LOOCV strategy. The best model (the ANN trained with 400 cycles) reached an overall accuracy of 90.7%, sensitivity of 89.9%, specificity of 98.2%, precision of 94.4, and an F1-score of 0.92, while avoiding false positives in three non-trained *emm*-types from the test set. Thus, we were able to develop a preliminary model capable of accurately predicting the four most-prevalent *emm*-types in our setting, including the highly virulent *emm*1, shown to be more associated with invasive disease. Our results demonstrate that, by reducing the time and cost of traditional methods, FTIR combined with ML is a viable and easily implementable alternative to sequencing-based methods for identifying GAS *emm*-types. Therefore, this strategy might be exploited in clinical practice to allow for a more rapid assessment of the potential risks of invasive infections and support the timely and correct choice of antibiotic and anti-inflammatory treatments.

## Figures and Tables

**Table 1:** Demographic and clinical characteristics of the strains.

Sample Name	Material	Isolation Date	*emm-*Type	*emm-*Subtype	Present in Previous Paper [20]	iGAS	Training/Test Dataset
iGAS_1	Blood culture	12 February 2023	1	1.0 (M1_global_)	Yes	Yes	Training
iGAS_2	CSF	6 April 2023	1	1.52 (M1_UK_)	Yes	Yes	Training
iGAS_3 †	Ear swab	19 April 2023	12	12.101	Yes	No	Test
iGAS_4 †	Skin swab	19 April 2023	89	89.0	Yes	No	Test
iGAS_5	Pleural fluid	19 April 2023	1	1.52 (M1_UK_)	Yes	Yes	Training
iGAS_6	Blood culture	11 December 2023	1	1.25 (M1_global_)	Yes	Yes	Training
iGAS_7	Blood culture	2 February 2024	1	1.0 (M1_UK_)	Yes	Yes	Training
iGAS_8 †	Synovial liquid	1 December 2023	75	75.0	Yes	Yes	Test
iGAS_9	Wound drainage	20 March 2024	3	3.93	No	Yes	Training
iGAS_10	Pus	4 April 2024	6	6.4	No	Yes	Training
iGAS_11	Venous blood culture	8 April 2024	1	1.3 (M1_global_)	No	Yes	Training
iGAS_12	Blood culture	4 April 2024	6	6.4	No	Yes	Training
iGAS_13	Venous blood culture	8 April 2024	1	1.3 (M1_UK_)	No	Yes	Training
iGAS_14	Pharyngeal swab	7 April 2024	1	1.0 (M1_global_)	No	Yes	Training
SGA_1	Pharyngeal swab	9 April 2024	4	4.19	No	No	Training
SGA_2	Vulvar swab	9 April 2024	3	3.93	No	No	Training
SGA_3	Pharyngeal swab	9 April 2024	3	3.93	No	No	Training
SGA_4	Vulvar swab	22 April 2024	6	6.0	No	No	Training
SGA_5	Anal swab	22 April 2024	4	4.19	No	No	Training
SGA_6	Pustule swab	20 April 2024	3	3.1	No	No	Training
SGA_7	Wound swab	20 April 2024	4	4.0	No	No	Training
SGA_8	Anal swab	17 May 2024	4	4.19	No	No	Training
SGA_9	Tracheal aspirate	2 May 2023	1	1.0 (M1_UK_)	No	No	Training
iGAS_16	Blood culture	20 October 2024	6	6.4	No	Yes	Training

† Strains not included in the training and validation of the model, which were only used as test sets.

## Data Availability

The original contributions presented in this study are included in this article/Supplementary Materials. Further inquiries can be directed to the corresponding author.

## Supplementary Materials

The following supporting information can be downloaded at https://www.mdpi.com/article/10.3390/diagnostics15233041/s1. Figure S1: Workflow of the study and potential clinical applications. Figure S2: Performance of the models on the test dataset. Table S1: Algorithms and parameters tested, with corresponding values.

## Abbreviations

The following abbreviations are used in this manuscript:

ANN	Artificial Neural Network
FTIR	Fourier-Transform Infrared Spectroscopy
GAS	Group A *Streptococcus*
iGAS	Invasive GAS infections
IR	Infrared
ML	Machine Learning
RBF	Radial Basis Functions
SVM	Support Vector Machine
