# Supplementary material for: Machine Learning Model with Fourier-Transform Infrared Spectroscopy (FTIR) as a Proof-of-Concept Tool for Predicting Group A Streptococcus (GAS) emm-Type in the Pediatric Population
Source: Diagnostics (Basel). 2025 Nov 28;15(23):3041. doi: 10.3390/diagnostics15233041 (PMC12691312; doi:10.3390/diagnostics15233041)
Supplement: Supplementary file 1 [file diagnostics-15-03041-s001.zip › diagnostics-3897083-supplementary/Supplementary_Figure_1.pptx]

## Slide 1
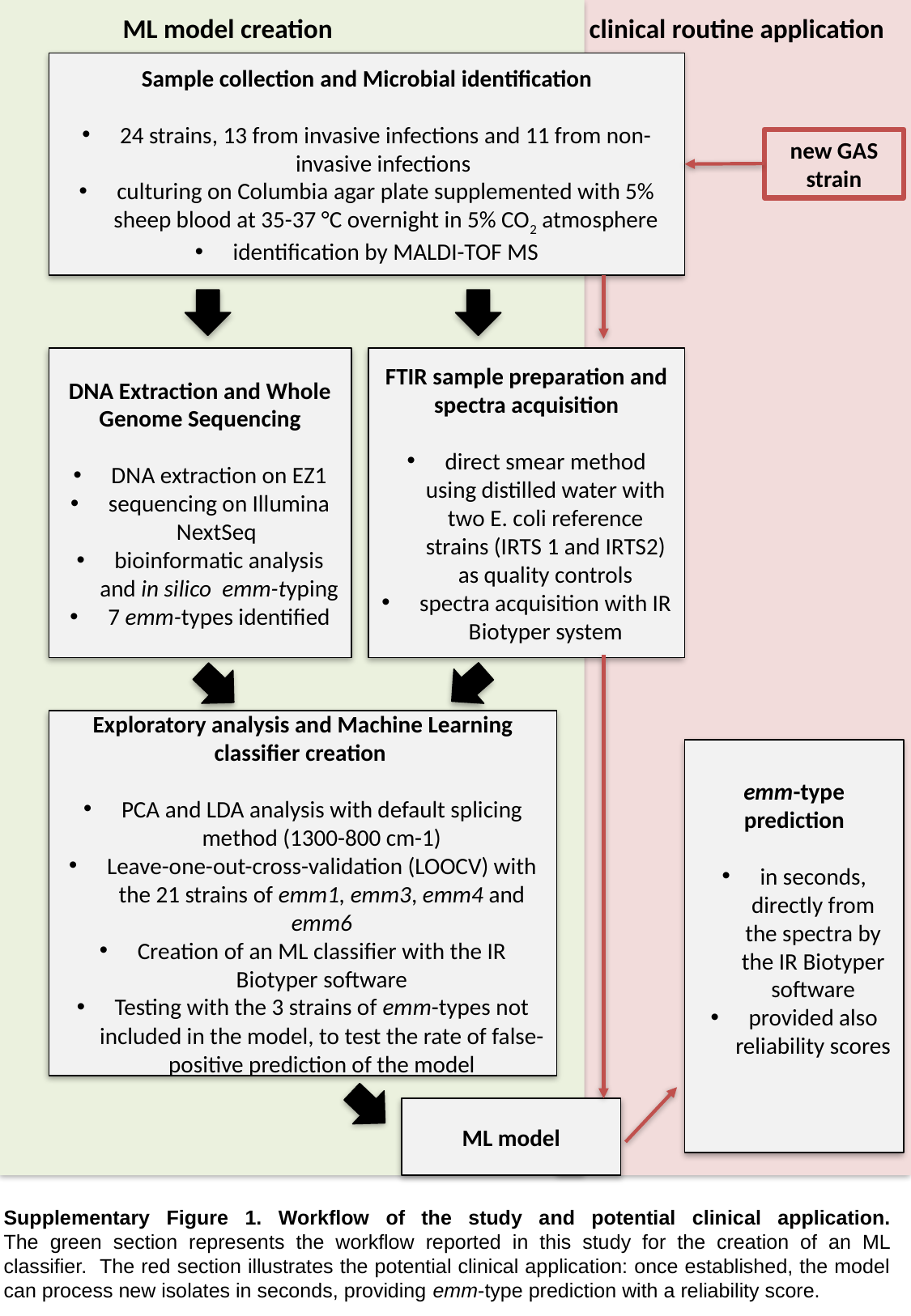

clinical routine application
ML model creation
Sample collection and Microbial identification
24 strains, 13 from invasive infections and 11 from non-invasive infections
culturing on Columbia agar plate supplemented with 5% sheep blood at 35-37 °C overnight in 5% CO2 atmosphere
identification by MALDI-TOF MS
new GAS strain
DNA Extraction and Whole Genome Sequencing
DNA extraction on EZ1
sequencing on Illumina NextSeq
bioinformatic analysis and in silico emm-typing
7 emm-types identified
FTIR sample preparation and spectra acquisition
direct smear method using distilled water with two E. coli reference strains (IRTS 1 and IRTS2) as quality controls
spectra acquisition with IR Biotyper system
Exploratory analysis and Machine Learning classifier creation
PCA and LDA analysis with default splicing method (1300-800 cm-1)
Leave-one-out-cross-validation (LOOCV) with the 21 strains of emm1, emm3, emm4 and emm6
Creation of an ML classifier with the IR Biotyper software
Testing with the 3 strains of emm-types not included in the model, to test the rate of false-positive prediction of the model
emm-type prediction
in seconds, directly from the spectra by the IR Biotyper software
provided also reliability scores
ML model
Supplementary Figure 1. Workflow of the study and potential clinical application.
The green section represents the workflow reported in this study for the creation of an ML classifier. The red section illustrates the potential clinical application: once established, the model can process new isolates in seconds, providing emm-type prediction with a reliability score.
