# Supplementary material for: Machine Learning Model with Fourier-Transform Infrared Spectroscopy (FTIR) as a Proof-of-Concept Tool for Predicting Group A Streptococcus (GAS) emm-Type in the Pediatric Population
Source: Diagnostics (Basel). 2025 Nov 28;15(23):3041. doi: 10.3390/diagnostics15233041 (PMC12691312; doi:10.3390/diagnostics15233041)
Supplement: Supplementary file 1 [file diagnostics-15-03041-s001.zip › diagnostics-3897083-supplementary/Supplementary_Figure_2.pptx]

## Slide 1
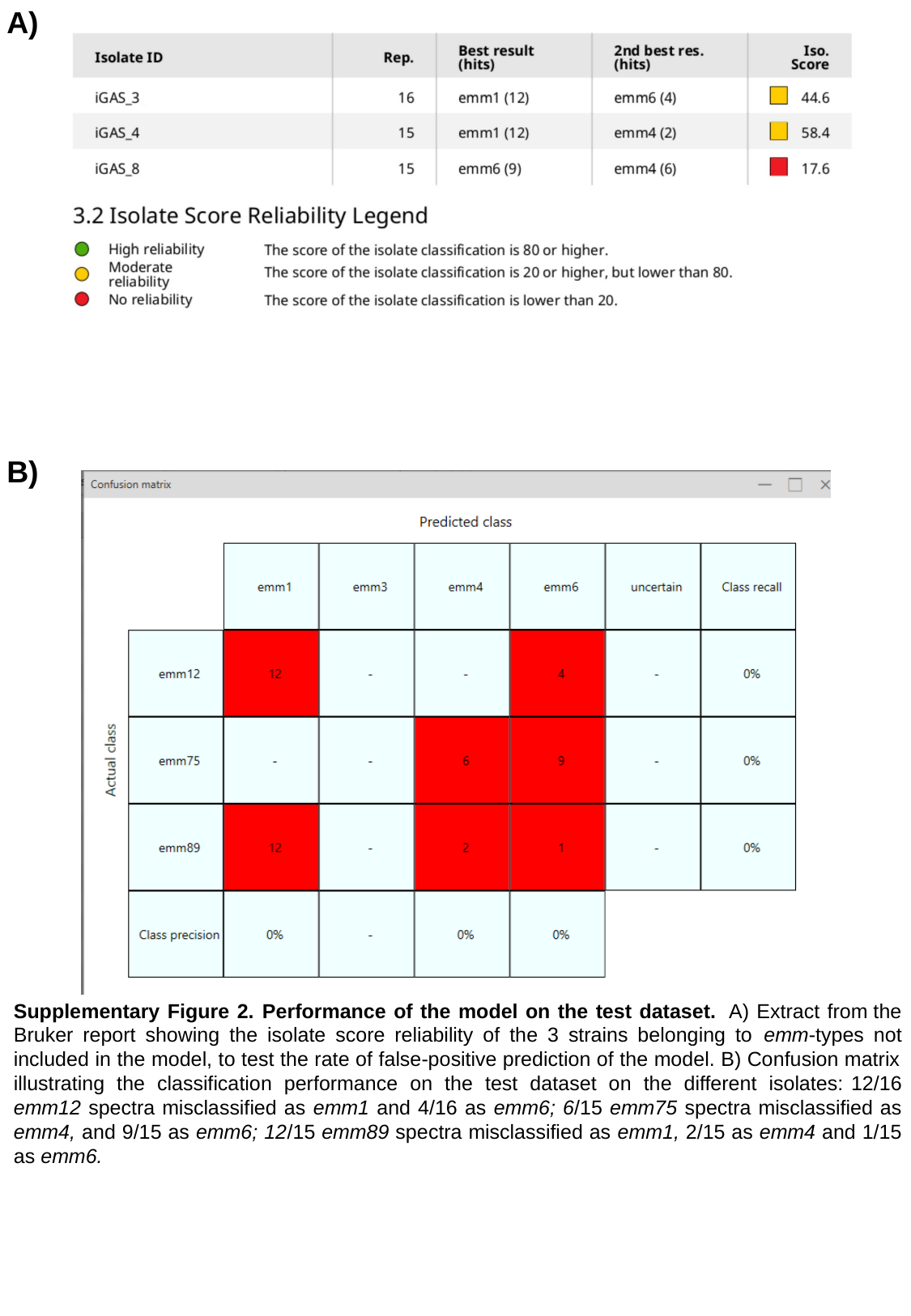

A)
B)
Supplementary Figure 2. Performance of the model on the test dataset.  A) Extract from the Bruker report showing the isolate score reliability of the 3 strains belonging to emm-types not included in the model, to test the rate of false-positive prediction of the model. B) Confusion matrix illustrating the classification performance on the test dataset on the different isolates: 12/16 emm12 spectra misclassified as emm1 and 4/16 as emm6; 6/15 emm75 spectra misclassified as emm4, and 9/15 as emm6; 12/15 emm89 spectra misclassified as emm1, 2/15 as emm4 and 1/15 as emm6.
